# Changing Face of the Gastroenterology Workforce: Progress and Pitfalls

**DOI:** 10.7759/cureus.107307

**Published:** 2026-04-18

**Authors:** Haleh Vaziri, Ohm Tripathi

**Affiliations:** 1 Gastroenterology and Hepatology, University of Connecticut, Farmington, USA; 2 Molecular and Cell Biology, University of Connecticut, Storrs, USA

**Keywords:** gastroenterology fellowship, gender equity, higher education medical training, minority groups, race and ethnicity

## Abstract

Background

Diversity in a health care system has many benefits, especially in underserved communities. Providing care to a diverse population by diverse physicians may translate to better compliance and outcomes. There is limited knowledge about ethnicity and gender representation in the gastroenterology (GI) workforce in the U.S. In this study, we aimed to evaluate the trends in racial and gender diversity in GI fellowships. We also examined the impact of COVID on the female gender and racial representation.

Methods

Accreditation Council for Graduate Medical Education (ACGME) data were queried to identify GI trainees between 2014 and 2024. Trainees were identified based on self-reported race and gender. We defined 2014-2020 and 2021-24 as pre- and post-COVID years, respectively. We also analyzed the Electronic Residency Application Service (ERAS) application data from 2015 to 2024 for race and gender. Pearson’s Chi-squared tests were used to compare proportions of female versus non-female fellows, and *t*-tests were used to compare the average Representation Quotients (RQ) within each racial group.

Results

We found a gender gap with the female fellows and male fellows representing 35.6% and 64.0% of the total, respectively (p<0.05). Female representation in GI fellowship increased significantly over the study period (2014-2015 to 2023-2024), with a notable rise observed in the post-COVID era compared with the pre-COVID era. The African-American population represented a lower proportion of GI fellows compared to the White and Asian populations. The Asian population had the highest representation in terms of female fellows, followed by White, Hispanic, and Black populations.

Conclusions

This study confirmed the disparities in gender and racial minority representation within ACGME-accredited GI fellowship programs. While there was an increase in the percentages of female fellows, the representation of the Black population remained stagnant.

## Introduction

Providing care to a diverse population by diverse physicians may translate to better compliance and outcomes. Despite the increased diversity in the United States (US) population in 2023 compared to 2014, racial disparities remain prevalent in both academic and clinical medicine in the US [1,2]. During the past several decades, the female population in the US has consistently been slightly larger than the male population. However, it has taken years for the medical school matriculant population to reflect these demographics [3]. Over the past several years, the number of women in medicine has steadily increased to the point that women now account for approximately half of all medical students in the US [4]. The increased number of female medical students has also translated into an increased number of women in residency programs, making up 45.64% of US residents (n=134,951) in 2019 [5]. While there has been an increase in the number of female trainees in Gastroenterology (GI) fellowship programs, this has not mirrored the increase observed in medical school and residency programs. In a retrospective study that evaluated data on the female participants in GI programs from 2009 to 2019, there was only a 3.3% increase in the representation of women during the 10 years, with an average of 33.6% of positions being filled by women [6].

In 2021, women represented 44.3% of internal medicine (IM) residents, 37.8% of GI fellows, and only 19.7% of practicing GI attendings. Since 2007, GI has consistently had one of the lowest proportions of women across all career stages among IM subspecialties, second only to cardiology [7]. With some female-predominant GI diseases [8-11] and female patients’ preference for female gastroenterologists/endoscopists [12,13], more female GI physicians are needed. In this study, we aimed to examine the trends in racial and gender diversity of trainees within GI fellowships and evaluate the impact of COVID on the racial and gender gap within GI training programs. 

This article was previously presented as a poster abstract at the 2025 American College of Gastroenterology Annual Scientific Meeting on October 27, 2025.

## Materials and methods

Data collection

Accreditation Council for Graduate Medical Education (ACGME) data were queried to identify GI trainees between 2014 and 2024. Trainees were identified based on self-reported race and gender. We defined gender groups as male subjects, female subjects, and others. Four specific racial groups were defined as White, Black/African-American, Hispanic/Latino, and Asian populations.

We defined 2014-2020 and 2021-24 as pre- and post-COVID years, respectively. Additionally, Electronic Residency Application Service (ERAS) application data for race and gender between 2015 and 2024 were also collected, using the same previously described definitions.

Statistical analysis

We aimed to assess trends over time in the overall number of GI fellows, applicants, self-reported gender (male, female, or non-binary/other), and racial sub-groups over 2014-2024. Linear regression was used to capture potential trends in the average number of total residents-per-program or total applicants over time. Pearson’s Chi-squared tests were used to compare proportions of female fellows versus non-female fellows, as well as the distribution of fellows within each previously described racial group, both per year and pooled (pre-COVID years vs. post-COVID). 

The four racial groups (White, Black/African-American, Asian, and Hispanic/Latino populations) were further analyzed using Representation Quotients (RQ), which quantified the degree of over- or under-representation of a specific group, versus the population average. RQs for a specific racial group were obtained by dividing the proportion of fellows in this group by the population proportion. In this study, we considered the population of interest to be US Medical School graduates - this data was obtained per-year, from the Association of American Medical Colleges (AAMC) [14]. For example, an RQ of 1.5 indicates that a specific group is 50% greater in the sample, compared to the overall population of medical school graduates. This approach has previously been used to assess trends in Urology applicants and matriculants, as well as other studies aiming to evaluate racial or gender-based trends [15,16]. After calculation of RQs per racial group, we assessed yearly and COVID-specific trends using linear regression models. We also used t-tests to compare the average RQ within each racial group to an RQ of one, which represents no over or under-representation. This analysis was repeated among ERAS applicants. 

RStudio version 4.4.1 (Posit, Boston, MA, USA) was used for all statistical analyses. All hypothesis tests were two-sided, with statistical significance considered at p<0.05.

## Results

Gastroenterology fellow data

Overall Trends

Between 2014 and 2024, data were collected on 17,523 GI fellows across an average of 200.6 programs per year. Annual fellow counts, program numbers, and mean fellows per program are presented in Appendix A. Both the total number of fellows and the total number of programs increased significantly over the study period (p<0.001 for each). In contrast, the mean number of fellows per program decreased slightly but significantly (linear regression estimate: −0.035 fellows per program per year post-2014; p=0.006), indicating that program expansion outpaced individual program growth.

Analysis of Gender

Across the study period, the average proportion of female GI fellows was 627.6 (35.8%), compared with 1117.4 (63.9%) male fellows (mean gender gap: 28.1; these figures represent averages of yearly proportions and are not paired with a single n value or percentage points). The proportion identifying as "Other" gender was negligible (average 0.4%) and was not independently analyzed. A significant increasing trend in representation in terms of female fellows was observed across the study period (Appendix A; Pearson's χ² = 42.7, p<0.001). Female representation increased from 503 (34.3%) in 2014-2015 to 815 (39.4%) in 2023-2024, peaking at 792 (40.1%) in 2022-2023 and reaching its nadir at 545 (32.9%) in 2017-2018. When pre-COVID years were compared with post-COVID years, female representation increased significantly (33.7% vs. 38.5%; Table 1).

**Table 1:** Gender distribution among gastroenterology fellows: Pre- vs. post-COVID comparison ^†^p-values derived from Pearson's chi-square tests comparing proportions pooled across pre-COVID (2014–2020) vs. post-COVID (2020–2024) periods.

Gender category	Pre-COVID Avg (%)	Post-COVID Avg (%)	Test statistic (χ²)^†^	p-value
Female	33.70%	38.50%	42.7	<0.001
Male	65.50%	61.40%	42.7	<0.001
Other	0.80%	0.10%	8.1	0.523

Analysis of Racial Subgroups

The average racial composition of GI fellows was: White participants (n=649.2; 37.0%), Asian participants (n=606.7; 34.2%), Hispanic/Latino participants (n=115.3; 6.4%), Black/African-American participants (n=85.4; 4.8%), and participants belonging to subgroups other than those mentioned earlier (Other; n=295.7; 17.6%). Detailed yearly proportions are shown in Appendix A.

Significant increasing trends across individual academic years were observed for Asian fellows (n=446; 30.4% in 2014-2015 to n=814; 39.4% in 2023-2024; χ² = 118.4, p<0.001) and Hispanic/Latino fellows (n=86; 5.9% to n=203; 9.8%; χ² = 58.6, p<0.001). When pre- and post-COVID periods were compared, fellows of Black/African-American, Asian, and Hispanic/Latino descent all increased significantly, while the proportion of White fellows remained unchanged (Table 2). 

**Table 2:** Distribution of different racial/ethnic populations among gastroenterology fellows: Pre- vs. post-COVID comparison ^†^p-values derived from Pearson's chi-square tests comparing proportions pooled across pre-COVID (2014–2020) vs. post-COVID (2020–2024) periods.

Race / Ethnicity	Pre-COVID Avg (%)	Post-COVID Avg (%)	Test Statistic; (χ²)^†^	p-value
White	36.70%	37.50%	11.3	0.316
Black/African-American	4.40%	5.50%	29.8	<0.001
Asian	30.30%	40.00%	118.4	<0.001
Hispanic/Latino	5.30%	8.20%	58.6	<0.001
Other	23.30%	8.80%	—	—

Average RQs for the primary analysis (including "Other population" in the denominator) were: White population 0.69, Black/African-American population 0.77, Asian population 1.56, and Hispanic/Latino population 1.16. Compared with an RQ of 1.0, fellows of White and Black/African-American descents were significantly under-represented relative to US medical school graduate proportions (p<0.001 for both), Asian fellows were significantly over-represented (p<0.001), and no significant disparity was detected for Hispanic/Latino fellows (p=0.099).

Because the category of "Other" population declined markedly over time (from 367 (25.0%) in 2014-2015 to 198 (9.6%) in 2023-2024), a secondary analysis excluding this group from the denominator was conducted. In this adjusted analysis, average RQs were: White population 0.84, Black/African-American population 0.94, Asian population 1.89, and Hispanic/Latino population 1.40. White fellows remained significantly under-represented (p<0.001); Asian and Hispanic/Latino fellows were significantly over-represented (p<0.001 and p=0.001, respectively); and no significant disparity was identified for Black/African-American fellows.

**Figure 1:**
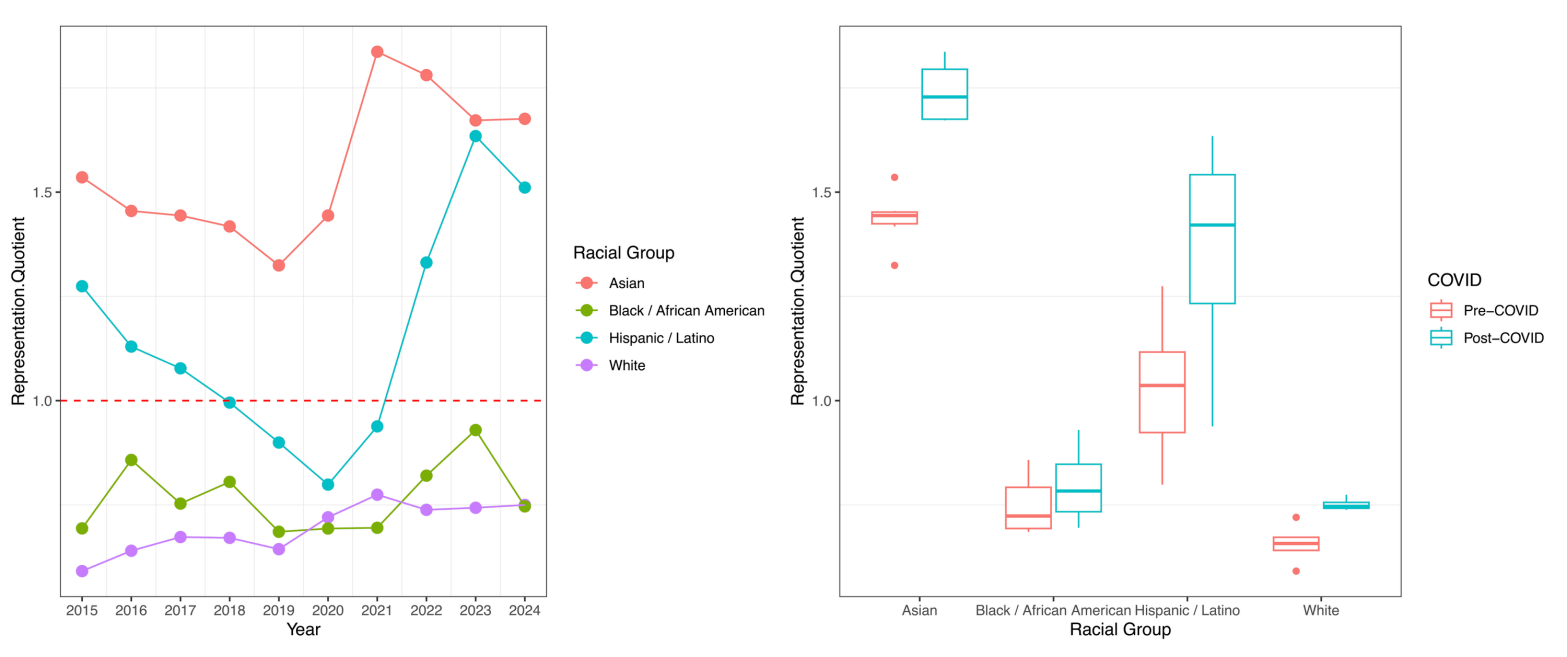
Longitudinal RQ trajectories and COVID-era averages of gastroenterology fellows RQ: Representation Quotient.

Linear regression of RQ over time identified a significant trend toward proportional representation for White fellows only (estimate: +0.017 RQ units per year post-2015; p<0.001). Pre-to-post-COVID comparisons showed significant RQ increases for White fellows (0.66 pre-COVID vs. 0.75 post-COVID; p=0.003) and Asian fellows (1.44 vs. 1.74; p<0.001). No significant RQ trends were detected for Hispanic/Latino or Black/African-American fellows. Longitudinal RQ trajectories and COVID-era averages are illustrated in Figure 1.

Gastroenterology applicant data

Overall Trends

A total of 9,944 GI fellowship applicants were included from 2014 to 2024. Annual applicant counts are presented in Appendix B. Total applicants increased significantly over time (linear regression estimate: +40.06 applicants per year post-2014; p<0.001).

Analysis of Gender

The average proportion of female applicants was 344 (34.4%, mean gender gap: 31.1 percentage points). Female applicants rose from 268 (31.6%) in 2014-2015 to 454 (39.0%) in 2023-2024, with the lowest proportion occurring in 2018-2019 at 294 (30.6%). Comparing pre- and post-COVID periods, female representation increased significantly (32.7% vs. 36.8%; Table 3).

**Table 3:** Gender distribution of gastroenterology applicants: Pre- vs. post-COVID comparison ^†^p-values derived from Pearson's chi-square tests comparing proportions pooled across pre-COVID (2014–2020) vs. post-COVID (2020–2024) periods.

Gender category	Pre-COVID Avg (%)	Post-COVID Avg (%)	Test statistic (χ²)^†^	p-value
Female	32.70%	36.80%	28.4	<0.001
Male	67.30%	63.20%	28.4	<0.001
Other	0.00%	0.00%	—	—

Analysis of Racial Subgroups

The average racial composition of the GI applicants was: White population (n=326; 32.8%), Asian population (n=327; 32.8%), Other population (n=223; 22.6%), Hispanic/Latino population (n=62; 6.2%), and Black/African-American population (n=56; 5.6%). Detailed yearly proportions are shown in Appendix B. Significant increasing trends across individual years were observed among Black/African-American applicants (n=50; 5.9% in 2014-2015 to n=79; 6.8% in 2023-2024; χ² = 9.8, p=0.023) and Hispanic/Latino applicants (n=52; 6.1% to n=89; 7.7%; χ² = 38.6, p<0.001). These trends were consistent in the pre- vs. post-COVID comparison (Table 4).

**Table 4:** Racial/Ethnic distribution of gastroenterology applicants: Pre- vs. post-COVID comparison ^†^p-values derived from Pearson's chi-square tests comparing proportions pooled across pre-COVID (2014–2020) vs. post-COVID (2020–2024) periods.

Race / Ethnicity	Pre-COVID Avg (%)	Post-COVID Avg (%)	Test statistic (χ²)^†^	p-value
White	32.20%	32.40%	0.8	0.762
Black/African-American	5.00%	6.50%	9.8	0.001
Asian	32.70%	33.50%	0.3	0.862
Hispanic/Latino	5.20%	7.60%	38.6	<0.001
Other	24.90%	20.00%	—	—

Average RQs for GI applicants were: White population 0.61, Black/African-American population 0.86, Asian population 1.50, and Hispanic/Latino population 1.11. Compared with an RQ of 1.0, White and Black/African-American applicants were significantly under-represented (p<0.001 and p=0.019, respectively). Asian applicants were significantly over-represented (p<0.001), and no significant disparity was identified for Hispanic/Latino applicants (p=0.148).

**Figure 2:**
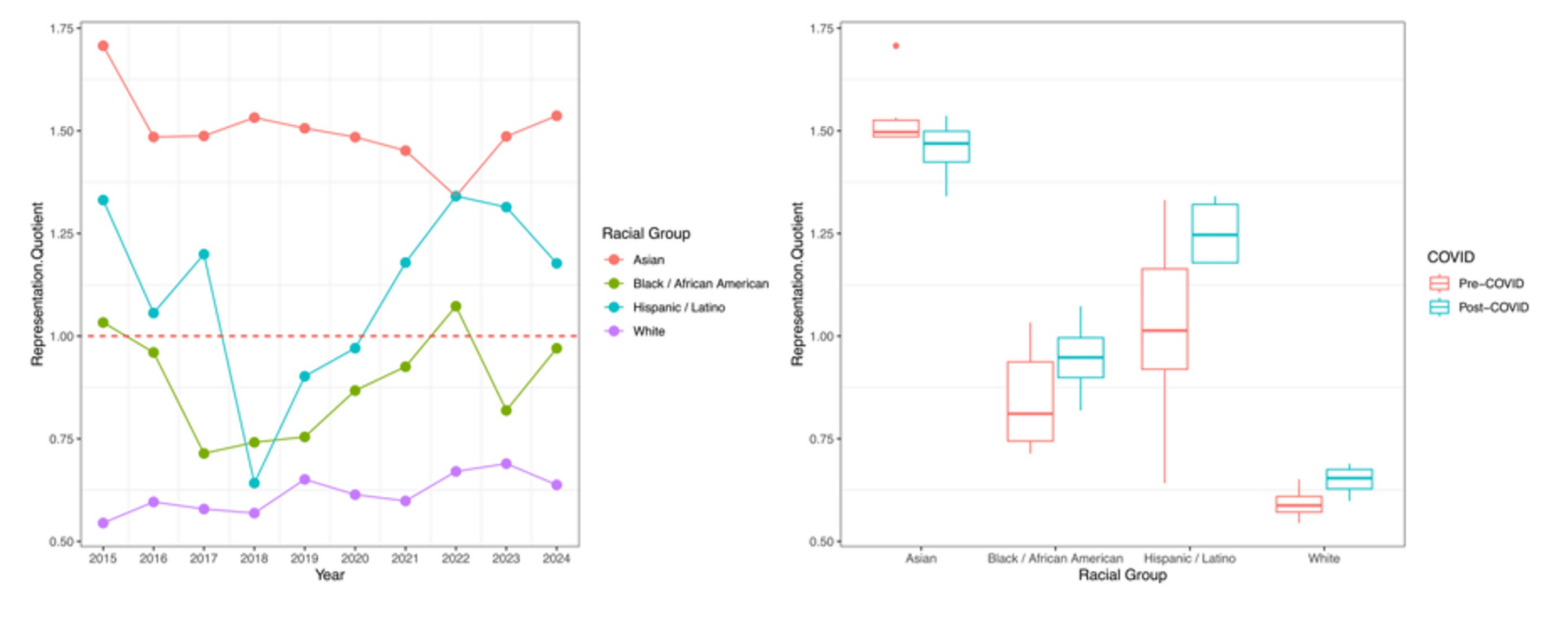
Representation Quotient trajectories and COVID-era averages for gastroenterology applicants

Linear regression of applicant RQ over time identified a significant trend toward proportional representation for White applicants only (estimate: +0.012 RQ units per year post-2015; p=0.006). No significant pre-to-post-COVID differences in RQ were detected for any racial group, although the comparison for White applicants approached significance (pre-COVID: 0.59 vs. post-COVID: 0.65; p=0.051). RQ trajectories and COVID-era averages for applicants are illustrated in Figure 2.

## Discussion

The importance of diversity in the medical field has been known for years. Patients generally achieve better outcomes when cared for by more diverse teams, with the additional benefit of improving innovation, team communication, and risk assessment within a diverse team [17]. Menees et al. [18] reported that 43% of women preferred a colonoscopy by a female endoscopist, 87% of those were willing to wait >30 days, and 14% would have paid more for a female endoscopist if needed. This gender preference in 75% of these patients was due to embarrassment. Five per cent of women would have declined colonoscopy unless performed by a female physician. 

Demographic factors, including age, ethnicity, and marital status, have previously been reported as barriers to career advancement for female clinicians compared to their male counterparts [19,20]. Gender-concordant mentorship plays an important role in professional development for early-career female gastroenterologists, providing much-needed guidance for work-life integration and gender-based challenges within the GI field [21,22]. Even though women are more likely to prefer same-sex mentors compared to men, a survey by the American Society for Gastrointestinal Endoscopy (ASGE) showed that the majority of female gastroenterologists had only male mentors. The presence of female faculty, especially in leadership positions, may also encourage more female candidates to apply to the fellowship program [6]. Pay inequality between men and women may be one of the major gender biases in GI [23-25], which may negatively affect the number of female trainees who apply to the field. 

In our study, we examined ACGME and ERAS data. The trends in the overall number of applicants, GI fellows, self-reported gender (male individuals, female individuals, or non-binary/other individuals), and racial sub-groups were evaluated from 2014 to 2024. We also assessed the impact of COVID-19 on the makeup of the GI fellowships. We found a growing number of GI fellowship programs (from 165 in 2014-15 to 238 in 2023-24) with an increase in the total number of GI fellows (from 1467 in 2014-15 to 2067 in 2023-24). It was also encouraging to see that the percentage of female fellows increased by 5.1% from 2014-15 to 2023-24 and the gender disparity decreased from 31% in 2014-15 to 21.1% in 2023-24. 

We also noticed that more female candidates were choosing GI in the post-COVID (38.5%) compared to the pre-COVID (33.7%) era (p=0.004). This significant demographic shift warrants further investigation. The transition to virtual fellowship interviews during the post-pandemic period may have influenced application patterns, potentially mitigating logistical barriers that historically impacted female candidates in the traditional in-person recruitment process. Another intriguing finding is that the rise in the female GI fellow percentage from 2020 to 2024 was the highest in the Hispanic population compared to any other race/ethnicity.

Diversity, equity, and inclusion (DEI) has remained a critical issue in medical fields, including GI, over the last several years. We observed a trend toward a higher proportion and number of Black and Hispanic applicants in more recent years. The significant drop in the “others” category after 2020-2021 compared to the prior years, might be due to better characterization of race. The lowest percentage of GI fellows were from the Black population (5.2%) compared to the White (36%), Asian (39.4%), and Hispanic (9.8%) populations in 2023-24. Analysis of the ERAS data revealed concerning patterns in racial demographics, with Black applicants representing the smallest proportion of GI fellowship candidates. Black population represents 13.7% of the current U.S. population, while Black fellows only accounted for 5.2% of total fellowships in 2023-24, resulting in a "racial-fellow gap" of 8.5%. Similarly, the U.S. Hispanic population is 19.5% of the general population [26], yet Hispanic fellows accounted for only 9.8% of the total, leaving a similar gap of 9.7%. The gap between Hispanic fellows (5.9%) and Black fellows (4%) has widened from 1.9% in 2014-15 to 4.6% (9.8% for Hispanic fellows vs. 5.2% for Black fellows) in 2023-24. This trend needs future research as one the representation for one under-represented minority has improved compared to the other. There are statistically more Black fellows post- vs. pre-COVID (p=0.024). Although the reasons behind this are unknown, the efforts of the GI societies to bring awareness to this issue and encourage the programs to recruit more under-represented populations in medicine can be one of factors contributing to this increase. At the same time, one may wonder whether changing the interview format from in-person to virtual, has made the interview process more accessible to minorities [27].

Unlike with gender, where fewer female participants entered GI fellowship programs compared to the number of women who got into medical schools from 2014-15 to 2023-24, certain minorities face a severely reduced inflow of graduates entering medical school, which correlates closely with their representation among IM residents. Despite GI societies’ efforts, there continues to be a gap between the representation of minority population in GI and the U.S. population [28]. Multiple factors may contribute to this under-representation, including the limited availability of racially concordant mentorship opportunities and accessibility to the interview process. Interestingly though, there is a rise in applicants of Hispanic descent from 52 in 2015 to 89 in 2024. 

Many medical schools have admissions policies to improve DEI as part of their mission or vision statements [29]. In 2019, the ACGME updated its common program requirements, asking the training programs to establish and implement strategies to recruit and retain individuals from under-represented medicine and medical leadership. Additionally, the ACGME specified that assessment of the diversity initiative should be a part of the program evaluations [30]. There is a vital need to increase workplace diversity, inclusion, and equity in medicine with efforts to advance women and minorities within the GI field and our societies are actively working toward this goal. 

Limitations

Our study has many strengths. It is the first of its kind to explore the effect of COVID-19 on the gender and racial distribution of GI fellows. It is also unique for looking at trends of female trainees among different races/ethnicities.

The study has some limitations that should be acknowledged. First, the data provided by the American Medical Association (AMA) and the AAMC are based on self-reports from fellowship programs, which may introduce inaccuracies. Second, the response rate from programs may not have been 100%, resulting in some trainees being unaccounted for. Additionally, a small number of respondents did not want to disclose their gender or race. Gender is not a binary issue, and we did not include a person who is gender neutral. We also need to be aware of the increasing number of people from mixed races in US demographics. Another limitation of the study is the aggregated nature of the data which limits our ability to adjust for confounding variables, such as location, when assessing trends over time or comparing racial and gender groups. Furthermore, we cannot provide insights into the reasons behind the persistently low numbers of Black and Hispanic members in the fellowships based on the retrospective data in our study. It should be acknowledged that comparing the number of ERAS applicants per race/ethnicity with the subsequent matched first-year fellows is essential to understand if racial trends are due to lack of diversity in the applicants and/or matching process. Unfortunately, this was not reported in our manuscript as National Resident Matching Program (NRMP) does not collect data on race and ethnicity of the matched individuals. Our data only provides a summary of diversity trends in GI fellowship programs without examining the reasons behind trainees’ choices for a specific subspecialty or the recruitment procedures that may have affected the outcome. Further research is necessary to address these limitations and to explore strategies for increasing diversity.

## Conclusions

Our study highlights meaningful progress in narrowing the gender gap within ACGME-accredited GI fellowship programs. The growing number of female candidates entering the GI field, with notable acceleration in the post-COVID era, reflects broader trends in medicine and is an encouraging sign for the specialty. These findings underscore the importance of continued efforts to support gender-concordant mentorship, address pay inequity, and foster inclusive recruitment practices to sustain this momentum.

Despite this progress, racial and ethnic representation remains an area of significant concern. Fellows of Black/African-American descent continue to be markedly under-represented relative to both the US population and medical school graduate proportions, and the widening disparity between representation of Hispanic and Black populations among GI fellows warrants focused investigation. Understanding the factors that influence these patterns will be important to ensure that future GI fellowship cohorts reflect the diverse US population.

## Appendices

Appendix A 

**Table 5:** Counts (% of the total) of US gastroenterology fellows (overall, and by race and gender) *Considered to be COVID year.

Year	Total programs	Total residents	Residents per program	Gender			Race/Ethnicity		
				Category	n	%	Category	n	%
2014–2015	165	1,467	8.89	Female	503	34.30%	White	510	34.80%
				Male	948	64.60%	Black/African-American	58	4.00%
				Other	16	0.90%	Asian	446	30.40%
							Hispanic/Latino	86	5.90%
							Other	367	25.00%
2015–2016	168	1,505	8.96	Female	506	33.60%	White	550	36.50%
				Male	986	65.50%	Black/African-American	71	4.70%
				Other	13	0.90%	Asian	462	30.70%
							Hispanic/Latino	85	5.60%
							Other	337	22.40%
2016–2017	175	1,565	8.94	Female	522	33.40%	White	595	38.00%
				Male	1,036	66.20%	Black/African-American	66	4.20%
				Other	7	0.40%	Asian	470	30.00%
							Hispanic/Latino	86	5.50%
							Other	348	22.20%
2017–2018	189	1,656	8.76	Female	545	32.90%	White	620	37.40%
				Male	1,105	66.70%	Black/African-American	76	4.60%
				Other	6	0.40%	Asian	493	29.80%
							Hispanic/Latino	89	5.40%
							Other	378	22.80%
2018–2019	202	1,741	8.62	Female	597	34.30%	White	612	35.20%
				Male	1,127	64.70%	Black/African-American	74	4.30%
				Other	17	1.00%	Asian	498	28.60%
							Hispanic/Latino	83	4.80%
							Other	474	27.20%
2019–2020	207	1,792	8.66	Female	600	33.50%	White	684	38.20%
				Male	1,178	65.70%	Black/African-American	82	4.60%
				Other	14	0.80%	Asian	577	32.20%
							Hispanic/Latino	83	4.60%
							Other	366	20.40%
2020–2021*	211	1,844	8.74	Female	681	36.90%	White	734	39.80%
				Male	1,162	63.00%	Black/African-American	91	4.90%
				Other	1	0.10%	Asian	762	41.30%
							Hispanic/Latino	109	5.90%
							Other	148	8.00%
2021–2022*	222	1,911	8.61	Female	715	37.40%	White	718	37.60%
				Male	1,195	62.50%	Black/African-American	105	5.50%
				Other	1	0.10%	Asian	769	40.20%
							Hispanic/Latino	145	7.60%
							Other	174	9.10%
2022–2023*	229	1,975	8.62	Female	792	40.10%	White	725	36.70%
				Male	1,181	59.80%	Black/African-American	123	6.20%
				Other	2	0.10%	Asian	776	39.30%
							Hispanic/Latino	184	9.30%
							Other	167	8.50%
2023–2024*	238	2,067	8.68	Female	815	39.40%	White	744	36.00%
				Male	1,251	60.60%	Black/African-American	108	5.20%
				Other	0	0.00%	Asian	814	39.40%
							Hispanic/Latino	203	9.80%
							Other	198	9.60%

Appendix B 

**Table 6:** Counts (% of the total) of US gastroenterology applicants (overall, and by race and gender) *Considered to be COVID years.

Year	Total Applicants	Gender			Race/Ethnicity		
		Category	n	%	Category	n	%
2014–2015	849	Female	268	31.60%	White	272	32.00%
		Male	581	68.40%	Black/African-American	50	5.90%
		Other	0	0.00%	Asian	287	33.80%
					Hispanic/Latino	52	6.10%
					Other	188	22.10%
2015–2016	852	Female	266	31.20%	White	290	34.00%
		Male	586	68.80%	Black/African-American	45	5.30%
		Other	0	0.00%	Asian	267	31.30%
					Hispanic/Latino	45	5.30%
					Other	205	24.10%
2016–2017	850	Female	275	32.40%	White	278	32.70%
		Male	575	67.60%	Black/African-American	34	4.00%
		Other	0	0.00%	Asian	263	30.90%
					Hispanic/Latino	52	6.10%
					Other	223	26.20%
2017–2018	923	Female	324	35.10%	White	293	31.70%
		Male	599	64.90%	Black/African-American	39	4.20%
		Other	0	0.00%	Asian	297	32.20%
					Hispanic/Latino	32	3.50%
					Other	262	28.40%
2018–2019	962	Female	294	30.60%	White	342	35.60%
		Male	668	69.40%	Black/African-American	45	4.70%
		Other	0	0.00%	Asian	313	32.50%
					Hispanic/Latino	46	4.80%
					Other	216	22.50%
2019–2020	1,048	Female	373	35.60%	White	341	32.50%
		Male	675	64.40%	Black/African-American	60	5.70%
		Other	0	0.00%	Asian	347	33.10%
					Hispanic/Latino	59	5.60%
					Other	241	23.00%
2020–2021*	1,050	Female	370	35.20%	White	323	30.80%
		Male	680	64.80%	Black/African-American	69	6.60%
		Other	0	0.00%	Asian	343	32.70%
					Hispanic/Latino	78	7.40%
					Other	237	22.60%
2021–2022*	1,099	Female	389	35.40%	White	375	34.10%
		Male	709	64.50%	Black/African-American	79	7.20%
		Other	1	0.10%	Asian	333	30.30%
					Hispanic/Latino	84	7.60%
					Other	228	20.70%
2022–2023*	1,148	Female	431	37.50%	White	391	34.10%
		Male	717	62.50%	Black/African-American	63	5.50%
		Other	0	0.00%	Asian	401	34.90%
					Hispanic/Latino	86	7.50%
					Other	207	18.00%
2023–2024*	1,163	Female	454	39.00%	White	356	30.60%
		Male	708	60.90%	Black/African-American	79	6.80%
		Other	1	0.10%	Asian	420	36.10%
					Hispanic/Latino	89	7.70%
					Other	219	18.80%
